# Supplementary material for: Cutaneous and acral melanoma cross-OMICs reveals prognostic cancer drivers associated with pathobiology and ultraviolet exposure
Source: Nat Commun. 2022 Jul 15;13:4115. doi: 10.1038/s41467-022-31488-w (PMC9287446; doi:10.1038/s41467-022-31488-w)
Supplement: Supplementary file 1 — Supplementary Information [file 41467_2022_31488_MOESM1_ESM.pdf]

Supplementary Information

Cutaneous and acral melanoma cross-OMICs reveals prognostic cancer drivers associated with pathobiology and ultraviolet exposure
Vicente A.L.S.A. *et al*.

Supplementary Figures

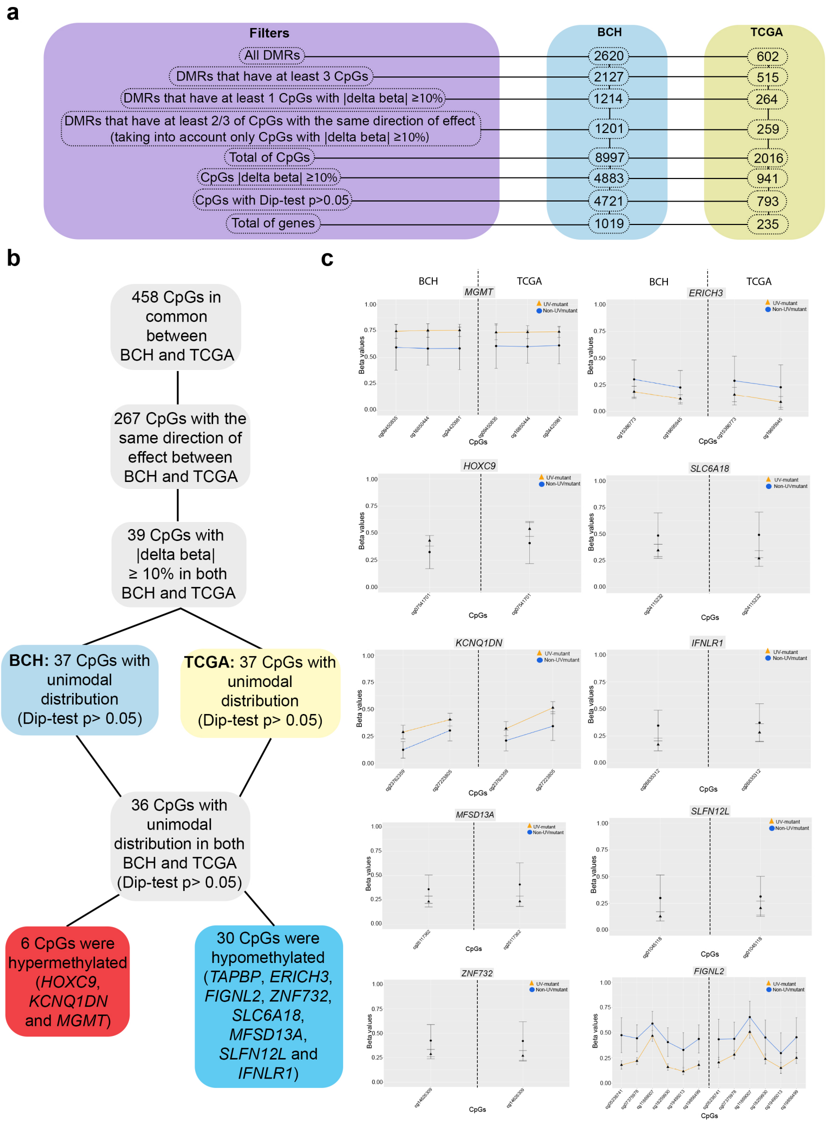

Supplementary Figure 1: Prioritization of UV methylation markers. a) Filtration steps applied to DMRs derived from the crude model in order to select the top CpGs and genes that are differentially methylated between UV-mutant *versus* non UV-mutant in BCH and TCGA. Significance was assessed using linear robust regression with FDR < 0.05. b) Prioritization criteria of UV-associated CpGs that are differentially methylated in both BCH and TCGA. c) DNA methylation levels of the 9 genes in common between BCH and TCGA showing differential methylation between UV-mutant (n= 44 and 47 in BCH and TCGA, respectively) and non UV-mutant patients (n= 44 and 47 in BCH and TCGA, respectively). Data were expressed as the average values of each group (UV-mutant and non UV-mutant) for each single CpG with error bars indicating the 95% confidence interval.

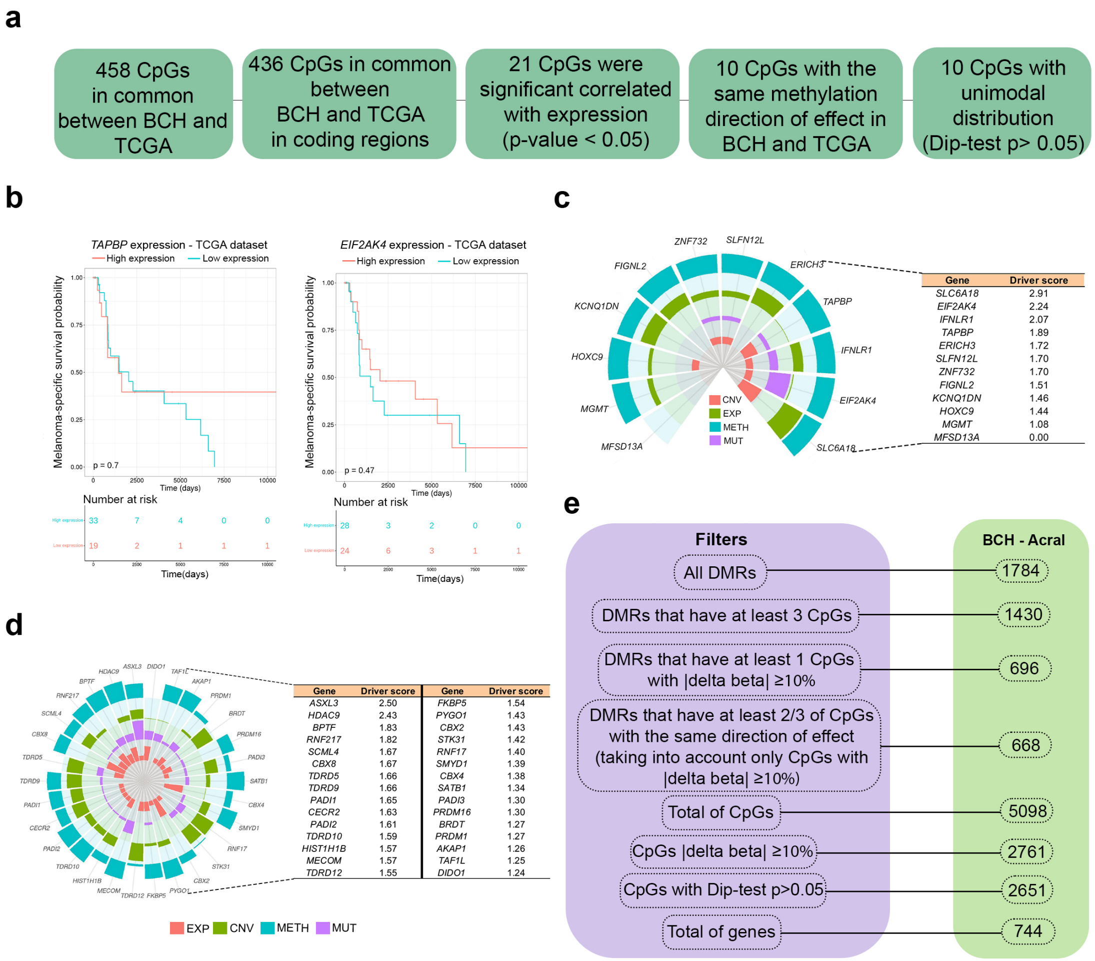

Supplementary Figure 2: Association of DNA methylation with transcription, transcription-mediated patient survival, cancer driver potential and pathobiology. a) Prioritization criteria of CpGs with significant expression quantitative trait methylation (eQTMs) ($p < 0.05$) in both BCH and TCGA. P-value was delivered from two-sided Pearson correlation and Dip-tests. b) Kaplan-Meier survival of melanoma patients in relation to expression levels of *TAPBP* and *EIF2AK4* measured in the target tumors derived from TCGA. Patients were categorized into low- and high-expression groups relative to the mean value of expression across profiled samples for a given gene. P values were derived by log-rank test. c) and d) Multi-omics data integration, encompassing copy number variation (CNV), expression (EXP), methylation (METH) and mutation (MUT), was performed in order to decipher the melanoma driver potential of the 12 prioritized genes (c) and of positive control genes previously identified in a recent study based on the ConsensusDriver method[1] (d). e) Filtration steps applied to DMRs derived from the crude model in order to select the top CpGs and genes that are differentially methylated between cutaneous and acral melanoma patients not harboring the UV mutational signature.

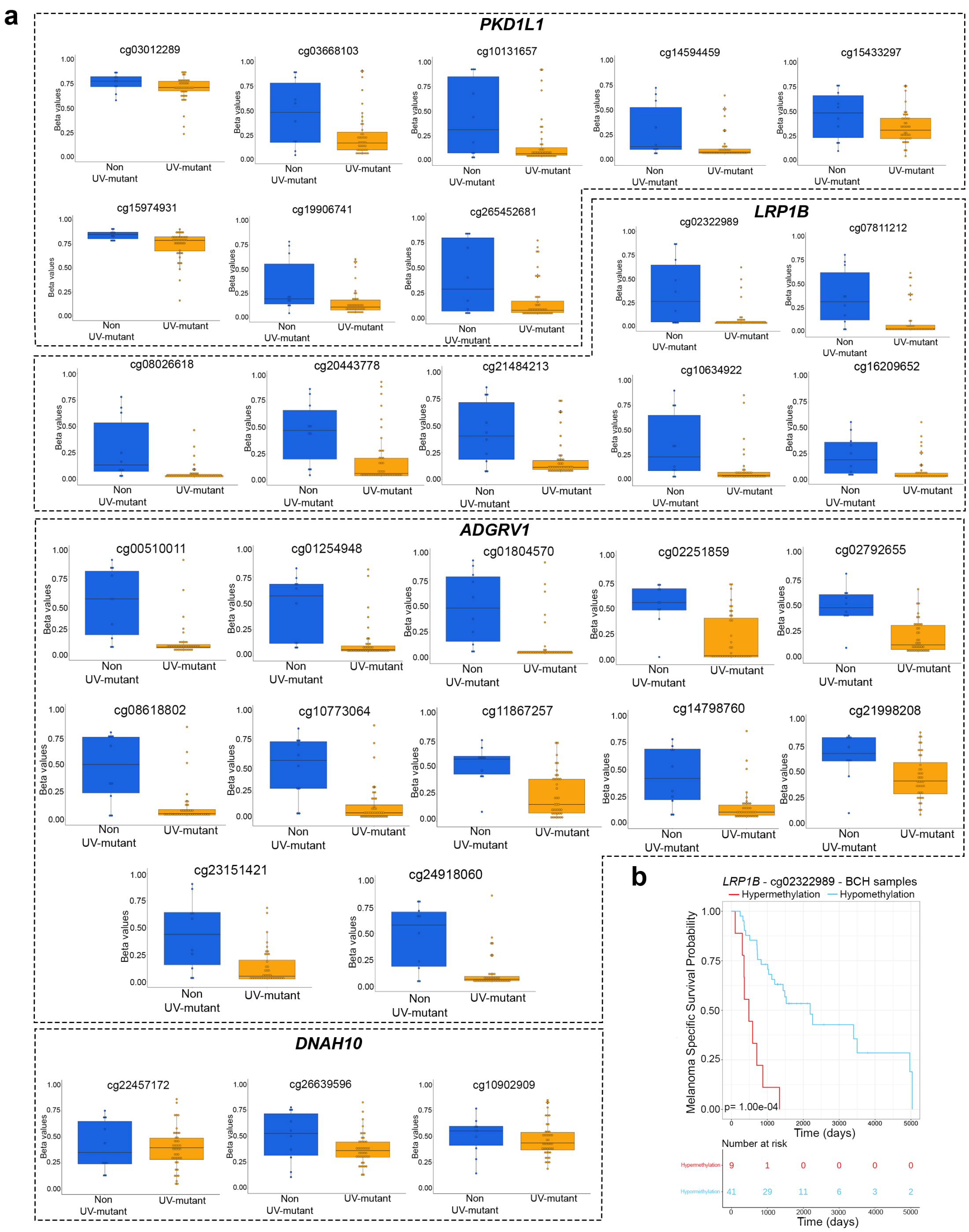

Supplementary Figure 3: DNA methylation alterations and clinical relevance of genes reported in the literature to be frequently mutated in UV-mutant *versus* non UV-mutant cutaneous melanoma patients[2]. a) *LRP1B*, *PKHD1L1*, *ADGRV1* and *DNAH10* were differentially methylated in UV-mutant (n= 44) relative to non UV-mutant (n= 10) patients in the BCH-cutaneous cohort. Box center lines, bound of the box, and whiskers indicate medians, first and third quantiles, and minimum and maximum values within 1.5xIQR (interquartile range) of the box limits, respectively. Each data point in the box plot represents the samples. b) Kaplan-Meier survival of cutaneous melanoma patients in relation to methylation levels of cg02322989 (*LRP1B*) measured in the target tumors derived from BCH. Patients were categorized into low- and high-methylation groups depending on whether the methylation value of a given CpG is lower or higher, respectively, than the mean methylation across the samples profiled for that CpG. The P-value was derived by log-rank test.

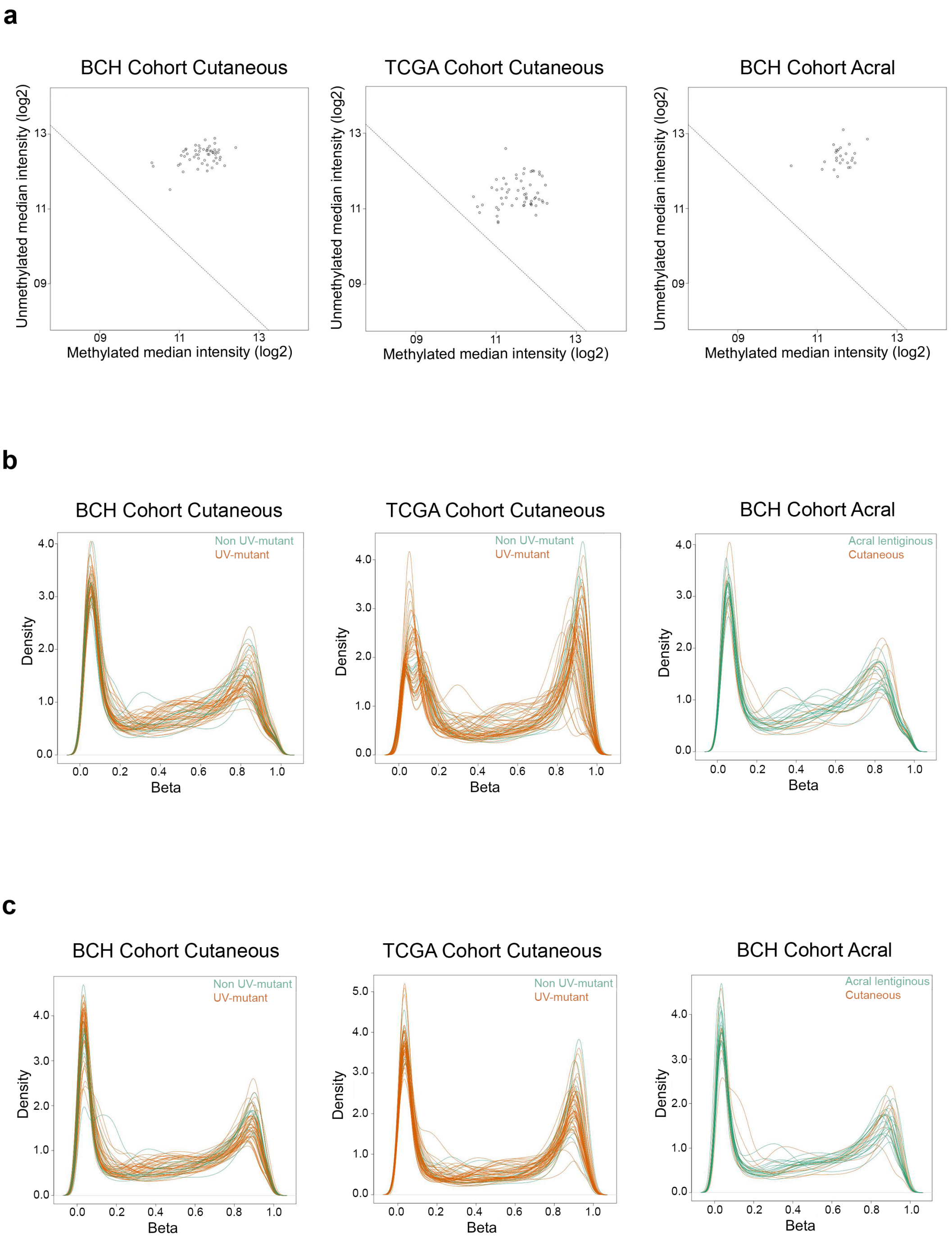

Supplementary Figure 4: Quality control of 450K methylation data. a) The plots show that all samples passed quality control (above diagonal threshold line) in each of the three indicated cohorts (BCH Cohort Cutaneous= 54, TCGA Cohort Cutaneous= 58 and BCH Cohort Acral= 21) ; b) Density plots of the beta methylation values for BCH-cutaneous, TCGA-cutaneous and BCH-acral datasets. In BCH-cutaneous and TCGA-cutaneous plots, orange and green densities represent samples harboring or not the UV signature, respectively. In BCH-acral, orange and green densities represent cutaneous (not harboring UV signature) and acral melanomas, respectively. c) Density plots of (b) after FunNorm normalization.

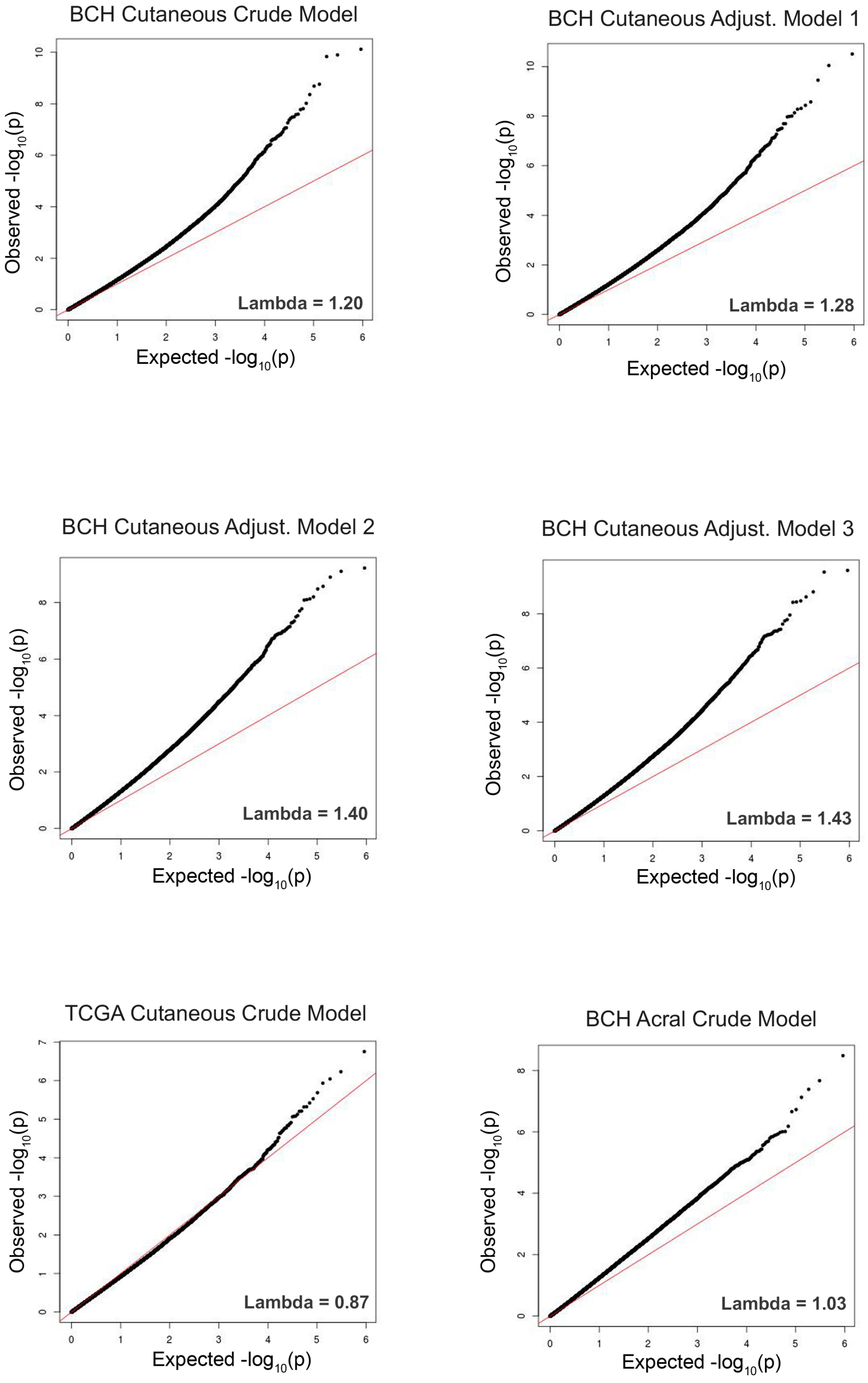

Supplementary Figure 5: Q-Q plots and lambda genomic inflation values of the statistical models used in BCH-cutaneous (n= 54), TCGA-cutaneous (n=58) and BCH-acral melanoma (n=17) cohorts. Crude and adjusted models are described in Methods.

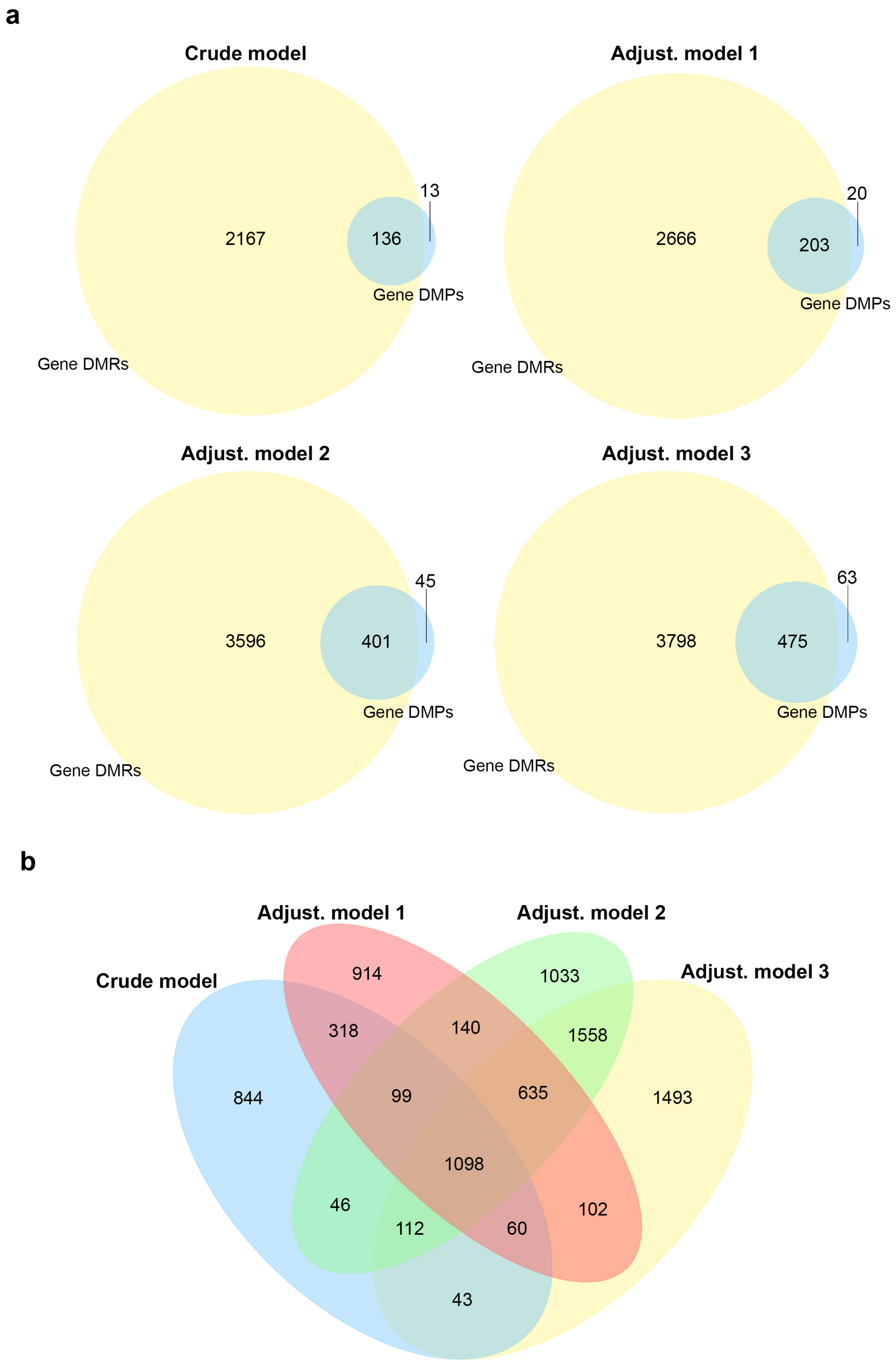

Supplementary Figure 6: Comparison of results yielded from the tested crude and adjusted statistical models as well as the two approaches used for the analyses, DMP and DMR. a) Approximately 90% of DMP-based genes overlapped with DMR-based genes across all tested models. b) Venn diagram showing DMR overlaps among the 4 statistical models.

Supplementary References

1. Bertrand, D. *et al.* ConsensusDriver Improves upon Individual Algorithms for Predicting Driver Alterations in Different Cancer Types and Individual Patients. *Cancer Res* **78**, 290-301 (2018).
2. Trucco, L.D. *et al.* Ultraviolet radiation-induced DNA damage is prognostic for outcome in melanoma. *Nat Med* **25**, 221-224 (2019).
