# Supplementary material for: Cutaneous and acral melanoma cross-OMICs reveals prognostic cancer drivers associated with pathobiology and ultraviolet exposure
Source: Nat Commun. 2022 Jul 15;13:4115. doi: 10.1038/s41467-022-31488-w (PMC9287446; doi:10.1038/s41467-022-31488-w)
Supplement: Supplementary file 3 — Description of Additional Supplementary Files [file 41467_2022_31488_MOESM3_ESM.pdf]

Description of Additional Supplementary Files

Cutaneous and acral melanoma cross-OMICs reveals prognostic cancer drivers associated with pathobiology and ultraviolet exposure
Vicente A.L.S.A. *et al*.

Supplementary Data 1. Systematic literature search conducted in the PubMed database until May 2021 in order to find out studies that investigated the DNA methylome profile of melanoma patients.

Supplementary Data 2. DMRs in relation to UV exposure in BCH-cutaneous crude model.

Supplementary Data 3. DMRs in relation to UV exposure in BCH-cutaneous adjusted for sex.

Supplementary Data 4. DMRs in relation to UV exposure in BCH-cutaneous adjusted for sex and age at diagnosis.

Supplementary Data 5. DMRs in relation to UV exposure in BCH-cutaneous adjusted for sex, age at diagnosis and tumor type (primary or metastatic).

Supplementary Data 6. DMRs in relation to UV exposure in TCGA-cutaneous crude model.

Supplementary Data 7. All CpGs differentially methylated comparing non UV-mutant *versus* UV-mutant in crude model that passed the filtration steps described in Supplementary Fig. 1a and used in pathway and heatmap cluster analysis (Figure 3a and 3c) in BCH.

Supplementary Data 8. All CpGs differentially methylated comparing non UV-mutant *versus* UV-mutant in crude model that passed the filtration steps described in Supplementary Fig. 1a and used in pathway and heatmap cluster analysis (Figure 3a and 3c) in TCGA.

Supplementary Data 9. Jensen Ontology analysis using genes prioritized in Supplementary Fig. 1a and described in Supplementary Data 7 in BCH cohort using Enrich-r website. P-value was delivered from two-sided Fisher's exact test.

Supplementary Data 10. Jensen Ontology analysis using genes prioritized in Supplementary Fig. 1a and described in Supplementary Data 8 in TCGA cohort using Enrich-r website. P-value was delivered from two-sided Fisher's exact test.

Supplementary Data 11. KEGG pathway analysis using genes prioritized in Supplementary Fig. 1a and described in Supplementary Data 7 in BCH cohort using Enrich-r website. P-value was delivered from two-sided Fisher's exact test.

Supplementary Data 12. KEGG pathway analysis using genes prioritized in Supplementary Fig. 1a and described in Supplementary Data 8 in TCGA cohort using Enrich-r website. P-value was delivered from two-sided Fisher's exact test.

Supplementary Data 13. KEGG Pathway analysis using CpGs prioritized in Supplementary Fig. 1a and described in Supplementary Data 7 in BCH cohort using missMethyl package, which adjusts for the number of CpG associated with each gene. P-value was delivered from two-sided Fisher's exact test.

Supplementary Data 14. KEGG Pathway analysis using CpGs prioritized in Supplementary Fig. 1a and described in Supplementary Data 8 in TCGA cohort using missMethyl package, which adjusts for the number of CpG associated with each gene. P-value was delivered from two-sided Fisher's exact test.

Supplementary Data 15. Differentially expressed genes comparing non UV-mutant and UV-mutant in TCGA cohort.

Supplementary Data 16. Jensen Ontology analysis using differentially expressed genes described in Supplementary Data 15 in TCGA cohort using Enrich-r website. P-value was delivered from two-sided Fisher's exact test.

Supplementary Data 17. KEGG Pathway analysis using differentially expressed genes described in Supplementary Data 15 in TCGA cohort using Enrich-r website. P-value was delivered from two-sided Fisher's exact test.

Supplementary Data 18. 458 CpGs in common between BCH and TCGA.

Supplementary Data 19. Meta-analysis of DMRs across the BCH and TCGA datasets considering FDR-adjusted $p < 0.05$ and DMRs with at least 3 CpGs. The approach used is fixed effects inverse variance-weighted meta-analysis. P-value was derived from two-sided test based on Z-score (obtained from the direction of effect and P-value observed in each DMR) and the standard normal cumulative distribution function.

Supplementary Data 20. Meta-analysis of DMRs across the BCH and TCGA datasets considering Bonferroni-adjusted $p < 0.05$ and DMRs with at least 3 CpGs. The approach used is fixed effects inverse variance-weighted meta-analysis. P-value was derived from two-sided test based on Z-score (obtained from the direction of effect and P-value observed in each DMR) and the standard normal cumulative distribution function.

Supplementary Data 21. Association between DNA methylation and melanoma-specific survival in BCH and TCGA cohorts of CpGs common between the two cohorts, after the filters applied in Supplementary Fig. 1b. P-values were derived from log rank test.

Supplementary Data 22. Primers used for pyrosequencing validation of the *TAPBP* gene. "For", "Rev" and "Seq" denote forward, reverse and sequencing primers, respectively.

Supplementary Data 23. Twenty-five most informative CpGs and transcripts using LASSO penalization for integrative analysis in the TCGA cohort.

Supplementary Data 24. DMRs in BCH cohort comparing acral *versus* cutaneous in non UV-exposed melanoma patients.

Supplementary Data 25. All CpGs differentially methylated comparing acral *versus* cutaneous BCH melanoma patients that passed the filtration steps described in Supplementary Fig. 2e and used in gene ontology analysis (Figure 6d).

Supplementary Data 26. Jensen Ontology analysis using genes prioritized in Supplementary Fig. 2e and described in Supplementary Data 25 in BCH-acral cohort using Enrich-r website. P-value was delivered from two-sided Fisher's exact test.

Supplementary Data 27. KEGG pathway analysis using genes prioritized in Supplementary Fig. 2e and described in Supplementary Data 25 in BCH-acral using Enrich-r website. P-value was delivered from two-sided Fisher's exact test.

Supplementary Data 28. KEGG Pathway analysis using CpGs prioritized in Supplementary Fig. 2e and described in Supplementary Data 25 in BCH cohort using missMethyl package, which adjusts for the number of CpG associated with each gene. P-value was delivered from two-sided Fisher's exact test.

Supplementary Data 29. Association between DNA methylation and melanoma-specific survival of genes frequently mutated in response to UV exposure[1]. P-values were derived from log rank test. Adjustment for multiple testing was done using FDR.

Supplementary Reference

1- Trucco, L.D. *et al.* Ultraviolet radiation-induced DNA damage is prognostic for outcome in melanoma. *Nat Med* **25**, 221-224 (2019).
